# Complete Genome Sequence of the Filamentous Fungus *Aspergillus westerdijkiae* Reveals the Putative Biosynthetic Gene Cluster of Ochratoxin A

**DOI:** 10.1128/genomeA.00982-16

**Published:** 2016-09-15

**Authors:** Alolika Chakrabortti, Jinming Li, Zhao-Xun Liang

**Affiliations:** a School of Biological Sciences, Nanyang Technological University, Singapore; b Department of Bioinformatics, School of Basic Medical Sciences, Southern Medical University, Guangzhou, Guangdong, China

## Abstract

Ochratoxin A (OTA) is a common mycotoxin that contaminates food and agricultural products. Sequencing of the complete genome of *Aspergillus westerdijkiae*, a major producer of OTA*,* reveals more than 50 biosynthetic gene clusters, including a putative OTA biosynthetic gene cluster that encodes a dozen of enzymes, transporters, and regulatory proteins.

## GENOME ANNOUNCEMENT

Ochratoxins are a group of mycotoxins predominantly produced by the filamentous fungi that belong to the genera *Aspergillus* and *Penicillium* (1, 2). This group of toxins is known to contaminate a variety of agricultural commodities and foods. Ochratoxin A (OTA), first isolated in 1965 from the culture broth of *Aspergillus ochraceus* (2), is one of the most-abundant food-contaminating mycotoxins. *Aspergillus westerdijkiae* is a mold fungal species that was initially assigned as a member of the *A. ochraceus* taxon. Unlike *A. ochraceus*, which does not consistently produce OTA, most isolates of *A. westerdijkiae* produce large amounts of OTA.

We sequenced the complete genome of *A. westerdijkiae* CBS112803 using the Illumina MiSeq platform and assembled the genome using SOAPdenovo at Macrogen Inc. in South Korea. The genome has a GC content of 50.35% and a genome size of 36.1 Mb. The genome size is comparable to those of several well-studied *Aspergillus* spp. such as *A. oryzae* (36.7 Mb), *A. fumigatus* (29.4 Mb), *A. nidulans* (30.6 Mb), and *A. niger* (34.0 Mb). The genome of *A. westerdijkiae* contains more than 50 secondary biosynthetic gene clusters according to antiSMASH version 3.0 (3), with the majority of them belonging to type I polyketide synthase (PKS) and nonribosomal peptide synthase (NRPS) gene clusters. Some of the gene clusters do not share significant similarity with the biosynthetic gene clusters from other *Aspergillus* spp., indicating that *A. westerdijkiae* has the potential to produce novel secondary metabolites.

The complete gene cluster for OTA biosynthesis remains unknown today, despite several existing reports on the involvement of PKS and NRPS in the biosynthesis. OTA contains a dihydroisocoumarin moiety that is most likely to be synthesized by a PKS (4, 5). A small portion of the *pks* gene for OTA biosynthesis in *A. ochraceus* has been reported previously (6). The *pks* genes (*otapksPN* and *AcOTApks*) involved in the production of OTA in *P. nordicum* and *A. carbonarius* have also been reported (7–9). By searching the homologous *pks* gene in the *A. westerdijkiae* genome, we identified a putative OTA biosynthetic gene cluster. The gene cluster spans 45.8 kb of DNA and encodes a dozen proteins that include several enzymes, regulatory proteins, and transporters. The enzymes encoded by the gene cluster include a type I iterative PKS with a KS-AT-DH-CMeT-ER-KR-ACP domain organization (KS, ketosynthase; AT, acyltransferase, DH, dehydratase; ACP, acyl carrier protein; KR, ketoreductase; ER, enoylreductase; CMeT, methyltransferase), an NRPS with an A-T-C-A-T domain organization (A, adenylation; C, condensation; T, peptidyl carrier protein), a flavin-dependent oxidoreductase, a cytochrome P450 monooxygenase, a halogenase, and a hydrolase. The presence of the cytochrome P450, oxidoreductase, and halogenase genes is consistent with the hydroxylation, oxidation, and chlorination steps needed to generate the final OTA product. The genomic information advances our understanding of OTA biosynthesis and paves the way for further elucidating the regulation of OTA production.

### Accession number(s).

The whole-genome sequence of *A. westerdijkiae* has been deposited in GenBank under the accession number LKBE00000000. The DNA sequences for the OTA genes can be found under the accession numbers LKBE01000001 to LKBE01000239.
